# History of Dentistry

**Published:** 1874-03

**Authors:** 


### HISTORY OF DENTISTRY.

There is in course of preparation a work to be entitled, "*The History of Dentistry in the United States.*" The design is to bring together in this volume everything that should be embraced in a complete and concise history, including an account of the profession in this country from its rise to the organization of the American Society of Dental Surgeons, and from that point continued by a presentation of the history of Dental Colleges—each one in particular; a history of each of the professional journals that is now, or has been, in existence, and of the textual and standard literature of the profession; also an account of Dental Societies; also of the various modes of practice that have been developed from time to time, the instruments, appliances and materials that have been and are used.

A full, accurate and reliable history will thus be presented so far as it is obtainable.

There will also be a biographical department, which will include sketches of prominent men of the profession who have passed away and of some living men who have been long in the profession.

The work will be a magnificent quarto of 500 pages or more, printed on handsome tinted paper, elaborately and profusely illustrated.

We trust that the volume will be one that no one who has any interest in the history of the dental profession can afford to be without; and as a matter of reference such a work would be invaluable. We shall occasionally make a note of the progress of the work. We shall be pleased to receive, from any one, an account of any matter that should be embraced in such a work.

